# 

**Published:** 1888-11

**Authors:** 


### BOOK NOTICES.

THE HOME-MAKER, is the title of a new monthly of 84 pp. devoted specially to "elevating the standard and increasing the efficiency of American Homes." The initial number of this new candidate for popular favor, is so replete with excellent home matters, as to give promise of great usefulness. Published by the Home Maker Company 24 West 23d. st. New York City, at $2 per annum.

DISINFECTION AND DISINFECTANTS.—This is a compilation of 266 pp. embodying the report of a committee composed of seven members prominent in the profession of medicine and surgery, appointed by the American Public Health Association at its annual session in 1884. " To examine the subject of disinfectants, antiseptics, and germicides in relation to preventive medicine and sanitation." If we are to form an opinion from the volume at hand, compiled by Dr. Irving A. Watson, the efficient secretary of the association, we should affirm that the committee have done their allotted work in a most complete and thorough manner, and Dr. Watson in sending out the volume in question, has contributed to the medical profession, a treatise of very great value. The descriptive drawings of the most approved buildings and mechanism employed in the processes of disinfection, add greatly to the importance of the work, whilst the treatises on the various bacteria and infusoria which are almost everywhere present to germinate disease, are the most complete we have anywhere seen. It is a work which should be in the hands of every physician in the active practice of his profession. For sale by Irving A. Watson Secretary, Am. P. H. Association, Concord N. H. Price $2, sent by post prepaid.

BLAKE'S WEATHER TABLES FOR 1889, is the title of a forthcoming book, intended as a weather-guide and forewarning to farmers for the ensuing year, suited to the different sections of the United States and Canada. The author is C. C. Blake, of Topeka, Kansas, who, for the past thirteen years, has devoted himself to meteorological calculations regarding the future, based upon strictly scientific problems, with which he has acquired great expertness. He predicts that the coming year will be remarkable for great extremes of temperature and unusual rainfalls.

TRANSACTIONS OF THE MEDICAL ASSOCIATION OF THE STATE OF MISSOURI, 1888—This is a most creditable volume extending to 462 pp. including the President's annual address, various essays and circumstantial reports of extraordinary cases of diseases and deformities, with a number of well executed illustrations. In hastily examining its contents we were led to remark that our western practitioners of the healing art, are not a whit behind their eastern brethern.

THE MURDOCK FREE SURGICAL HOSPITAL FOR WOMEN, founded and supported solely by Albert S. Murdock, Boston, is one of the very best charitable institutions in this country, the annual report of which is before us, showing that 548 patients have shared its benefits.

THE KINDERGARTEN.—A monthly publication in pamphlet form, and excellently well done in matter and style, is published at 161 La Salle st., Chicago, Alice B. Stockham & Co., publishers.

We have always advocated the Kindergarten system of instructing the very young, as the only one suited to them, and we are glad to know that it now has a fit organ in the one referred to.

THE CENTURY for November is, as usual, excellent. We do not wonder at its wide circulation. The Century Company, New York City.

YOUTH'S COMPANION.—The very best paper for all ages and sizes in existence.

